# Reduction of radiation exposure and preserved image quality using photon-counting detector cardiac computed tomography without electrocardiographic gating in children with congenital heart disease

**DOI:** 10.1007/s00330-025-11719-6

**Published:** 2025-07-03

**Authors:** Susanne Hellms, Thomas Werncke, Joachim Böttcher, Christoph M. Happel, Jan Eckstein, Markus Benedikt Krueger, Christoph Panknin, Alexander Pfeil, Till F. Kaireit, Philipp Beerbaum, Jens Vogel-Claussen, Frank Wacker, Diane Miriam Renz

**Affiliations:** 1 Institute of Diagnostic and Interventional Radiology, Hannover Medical School, Hannover, Germany; 2 Friedrich-Schiller-University Jena, Jena, Germany; 3 Clinic for Pediatric Cardiology and Intensive Care, Hannover Medical School, Hannover, Germany; 4 Siemens Healthineers, Forchheim, Germany; 5 Department of Internal Medicine III, University Hospital Jena, Jena, Germany

**Keywords:** Tomography (X-ray computed), Heart defects (Congenital), Radiation dosage, Pediatric

## Abstract

**Objectives:**

To evaluate the radiation exposure, quantitative, and qualitative image quality in pediatric cardiac CT by using photon-counting detector computed tomography (PCD CT) versus energy-integrating detector CT (EID CT) in matched children.

**Materials and methods:**

Thirty-seven contrast-enhanced, clinically indicated cardiac CTs performed on PCD CT were matched with 37 examinations acquired by EID CT. The patients were matched according to water-equivalent diameters. Quantitative evaluation of image quality comprised a region of interest (ROI)-based analysis, calculating image noise, signal-to-noise (SNR) and contrast-to-noise (CNR) ratio. Differences of the attenuation variation of the paraspinal and the pectoral muscles were calculated to measure beam hardening artifacts. Volume CT dose index (CTDI_vol_) and dose length product (DLP) were documented, and the effective radiation dose was calculated for each patient. Statistical analysis comprised *t*-tests and Wilcoxon signed rank tests.

**Results:**

The mean age of the children on PCD CT was 794 ± 1016 days, similar to the mean age of 815 ± 957 days of the children on EID CT (*p* = 0.76). Moreover, age, height, weight, and body mass index (BMI) were also not significantly different between the two groups (*p* ≥ 0.32). Radiation exposure was significantly lower on PCD CT (CTDI_vol_ 0.20 ± 0.12 mGy and DLP 4.06 ± 3.22 mGy*cm) versus EID CT (CTDI_vol_ 0.37 ± 0.17 mGy*, p* < 0.001 and DLP 7.21 ± 4.67 mGy*cm*, p* < 0.001). No significant differences in SNR, CNR, or beam hardening artifacts could be observed. Qualitative image quality was also comparable for PCD CT versus EID CT.

**Conclusions:**

With a reduction in radiation exposure exceeding 40% by using PCD CT, image quality remained stable compared to EID CT. Reducing radiation with PCD CT while preserving image quality might substantially advance cardiac imaging in children.

**Key Points:**

***Question*** *Children are particularly sensitive to radiation exposure, highlighting the need for dose reduction*.

***Findings*** *Radiation dosage can be significantly reduced while preserving image quality when using photon-counting detector (PCD) CT in pediatric patients with congenital heart disease*.

***Clinical relevance*** *Since radiation exposure can be significantly reduced by PCD CT compared to energy-integrating detector (EID) CT, while image quality was comparable, PCD CT is advisable for children with congenital heart disease*.

**Graphical Abstract:**

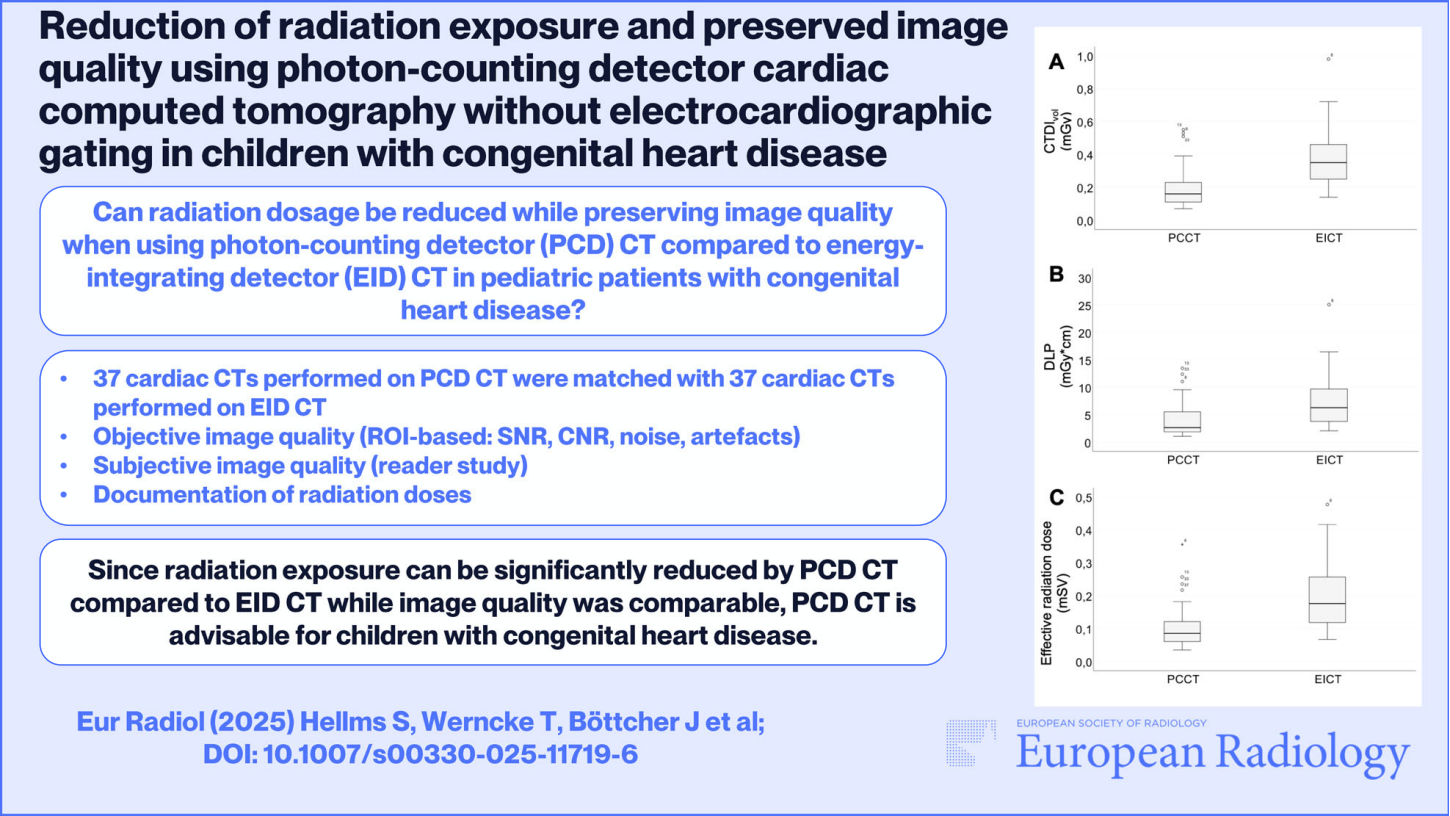

## Introduction

Cardiac CT has become a clinical routine in adult patients and increasingly in children [1–4]. Imaging the heart in small children and neonates represents a challenge due to their small, often intricate congenital anatomy. Moreover, their fast respiration and heart rates pose a high risk for pulsation and movement artifacts [5, 6]. Major clinical indications for pediatric cardiac CT are completion of initial diagnosis and pre-interventional/pre-surgical planning in patients with congenital heart defects, which are a leading cause for morbidity and mortality in the neonatal period, with an incidence of up to 1% of live births [7–9]. Scan times of CT examinations are extremely short, which is crucial in pediatric patients. The major drawback is the need for ionizing radiation. Previous studies have highlighted the need to reduce radiation exposure in patients with congenital heart disease, as the lifetime risk of cancer is increased [10, 11].

Photon-counting detector CT (PCD CT) is an emerging method as an alternative to conventional CT scanners. Conventional CT scanners use energy-integrating detectors where each detector element absorbs arriving X-rays that are then converted into visible light [12, 13]. Thus, an electrical signal, proportional to the amount of visible light that is measured by a photodiode, is generated, rather than the energy of an individual X-ray photon [11, 12], while photon-counting detectors convert individual X-ray photons directly into electric signals [12–14]. Compared to energy-integrator detector CT (EID CT), PCD CT has been described to offer improved spatial and contrast resolution, less electronic noise, less blooming, metal and beam-hardening artifacts, and increased iodine signal after contrast medium application [6, 12–18]. Due to the possible benefits, PCD CT has the potential to address the challenges of cardiac CT, including the need for a high spatial and contrast resolution [19–22].

Publications concerning the use of pediatric cardiac imaging by PCD CT are sparse. A recent study showed that higher signal-to-noise ratio (SNR) and contrast-to-noise ratio (CNR) could be achieved by PCD CT compared to EID CT at a similar radiation dose in children suspected of having congenital heart defects [23]. Patients examined on PCD CT versus EID CT were not matched, but consecutively enrolled [23].

The purpose of our study was to compare quantitative and qualitative image quality as well as radiation dose in propensity-matched pediatric patients, comparing a PCD CT versus an EID CT system. It is hypothesized that the imaging qualities between PCD CT and EID CT remain consistent, while PCD CT achieves a significant reduction in radiation dose.

## Materials and methods

### Study population

The retrospective study was conducted according to the guidelines of the Declaration of Helsinki and local data protection guidelines. The study was approved by the local institutional review board (institutional review board number: 10679_BO_K_2022). Informed consent was waived due to the retrospective study design, in accordance with the local institutional review board and data protection guidelines. Children, aged between 0 and 10 years, with congenital heart disease and clinically indicated cardiac CT examinations were included in order for initial diagnosis and/or prior to surgery/intervention with cardiac catheterization. The CT technical protocol was standardized and adapted between the two CT scanners (for details, see Table 1). Within a consecutive time period of 39 months, 45 pediatric patients were examined on PCD CT and 147 pediatric patients on EID CT; if the patients received more than one CT examination within the time period, only the first CT scan was included to reduce patient bias. A further exclusion criterion was the presence of metal devices visible on the localizer radiograph, as the radiation exposure increases due to automatic dose regulation. These metallic devices were diverse, ranging from small stents, clips or metallic sternotomy sutures to cardiac pacemakers. To ensure greater homogeneity within patient groups, patients with all metallic devices visible on the localizer radiograph were excluded from the analysis. This criterion resulted in the exclusion of 8 patients, who underwent PCD CT, and 23 patients with CT examinations on EID CT. The remaining 37 patients, performed on PCD CT, were matched with 124 patients, examined on EID CT, according to best matches for water-equivalent diameters. The calculation of water-equivalent diameters to accurately assess patient size in CT was performed, as described previously [24, 25]. Thus, 37 patients examined on PCD CT were compared to 37 matched patients with CT examinations on EID CT.

**Table 1:** Acquisition and reconstruction parameters for PCD CT and EID CT examinations

Parameter	PCD CT	EID CT
Tube voltage (kV)	70	70
Automated exposure control	Yes	Yes
Reference mAs/image quality level	45^#^	242^+^
Pitch	3.2	3.0
Gantry rotation time (s)	0.25	0.25
Detector collimation (mm)	144 × 0.4	192 (2 × 96) × 0.6
Matrix	768 × 768	512 × 512
Reconstructed slice thickness/increment (mm)	1.0/0.7	1.0/0.7
Convolution kernel	Br40	Bv40
IR algorithm	QIR	ADMIRE
IR strength	2	2

*PCD CT* photon-counting detector computed tomography, *EID CT* energy-integrating detector computed tomography, *IR* iterative reconstruction, *QIR* quantum iterative reconstruction, *ADMIRE* advanced modeled iterative reconstruction

^#^ Reference mAs control parameter was the CARE keV Image Quality level

^+^ Reference mAs control parameter was the Quality Reference mAs

### CT acquisition and reconstruction

Thirty-seven cardiac CT studies were performed on a first-generation clinical photon-counting detector CT system (NAEOTOM Alpha, Siemens Healthineers), and 37 cardiac CT examinations were acquired on a last-generation dual-source CT scanner using an energy-integrating detector (SOMATOM Force, Siemens Healthineers). All CT examinations were performed with the patient in supine position with the arms elevated in a craniocaudal scanning direction, capturing the whole thorax and heart. The cardiac CT examinations were performed in free-breathing, without electrocardiogram (ECG)-triggering, but with bolus tracking technique after weight-adjusted intravenously injected contrast medium (iomeprol; Imeron 400, Bracco Imaging), followed by saline flush. The contrast medium protocol was set on a fixed dose of 7 mL iomeprol for children between 3 and 6 kg body weight and a weight-adapted dose of 1 mL/kg body weight iomeprol for children with ≥ 7 kg body weight. Children < 3 kg body weight were not part of the study collective. The CT scans were started at the time point when all four heart chambers were optimally filled with contrast medium. The optimal time point was visually identified by experienced technicians.

The images were acquired as fast high-pitch helical scans on both CT systems. As the average examination time was less than a second, the CT studies were performed without sedation in older children and with sedation in small children; no general anesthesia was administered. Detailed technical parameters are summarized in Table 1. For quantitative and qualitative image analyses, images were reconstructed with a raw-data-based, advanced modeled iterative reconstruction level 2 in 1-mm slice thickness with an increment of 0.7 mm. The reconstruction time was between 20 and 30 s for both CT scanners. In a preliminary analysis, three independent raters determined that the convolution kernels Br40 (PCD CT) and Bv40 (EID CT) provided the most comparable image quality. Thus, these convolution kernels were used in the quantitative and qualitative image analysis to provide a blinded evaluation concerning the CT scanner type.

### Evaluation of radiation exposure

Radiation exposure of each CT examination was recorded. These parameters comprised volume CT dose index (CTDI_vol_) and dose length product (DLP). Effective radiation dose was calculated, as described previously [26], by using the following method: dose length product × age-related organ weighting factor = effective radiation dose. Weighting factors for chest CT in children, which have been published by the International Commission on Radiological Protection, were used, being 0.032 for children under the age of 5 and 0.019 for children between 5 and 10 years old [27].

### Quantitative evaluation of image quality

Quantitative image quality parameters were determined in a region-of-interest (ROI)-based analysis. To determine signal-to-noise ratio (SNR) and contrast-to-noise ratio (CNR), circular ROIs were manually placed on axial images in the tracheal air column above the tracheal bifurcation and in the main pulmonary artery [28]. ROI size was adapted individually to the diameter of the trachea and the pulmonary artery, respectively, to include the largest area possible without measuring adjacent tissues. The standard deviation (SD) of the CT attenuation within the ROI inside the trachea was defined as image noise [28]. According to Braun et al [28], contrast in chest CT is largely due to the difference in attenuation between pulmonary vasculature and surrounding parenchyma. SNR and CNR were calculated as follows:

- SNR = Mean CT attenuation in pulmonary trunk/image noise inside the trachea,
- CNR = (Mean CT attenuation in pulmonary trunk − mean CT attenuation in air inside the trachea)/image noise inside the trachea.

Effects of beam-hardening and electronic noise artifacts were quantified based on objective noise, which was defined as the SD of the measured density [29, 30]. ROIs were placed in the paraspinal muscles at the level of the aortic arch, a typical location affected by beam hardening due to the vertebrae and the scapulae (N1), and in the pectoral muscle, usually unaffected by beam hardening (N2) [29]. Beam-hardening artifact (BHA) was calculated as follows [31]:

$${{\rm{BHA}}}=\sqrt{{N1}^{2}-{N2}^{2}}.$$

### Qualitative evaluation of image quality

CT studies were analyzed in consensus by two experienced readers (> 15 years’ experience in cardiac CT), in a blinded manner without knowledge of the scanner type. Homogeneity of contrast, sharpness, overall image quality, and presence of pulsation artifacts were rated using a 5-point Likert scale as follows: 5 = excellent, no artifacts; 4 = good, mild artifacts; 3 = assessable, moderate artifacts; 2 = limited, severe artifacts; 1 = not assessable [32–34]. Furthermore, the image quality of four proximal segments of the coronary arteries was assessed: left main artery (LM), proximal left anterior descending artery (LAD), proximal left circumflex artery (LCX), and proximal right coronary artery (RCX) [35]. The proximal segments were assessed as follows: 5 = clear visualization without any motion artifacts, 4 = mild motion artifacts but high diagnostic confidence, 3 = obvious blurring, moderate diagnostic confidence, 2 = identified but equivocal, may simulate other structures, 1 = severe blurring, no coronary artery segment can be visualized (no useful information obtained) [35].

### Statistical analysis

Statistical analysis was performed using SPSS software version 28 (IBM). Data was tested for normal distribution according to the Shapiro–Wilk test. Non-parametric (Wilcoxon signed rank tests) and parametric (paired *t*-tests) tests were chosen accordingly. *p*-values < 0.05 were considered statistically significant. Values are given as mean ± SD if not indicated differently.

## Results

### Patient characteristics

**Fig. 1:**
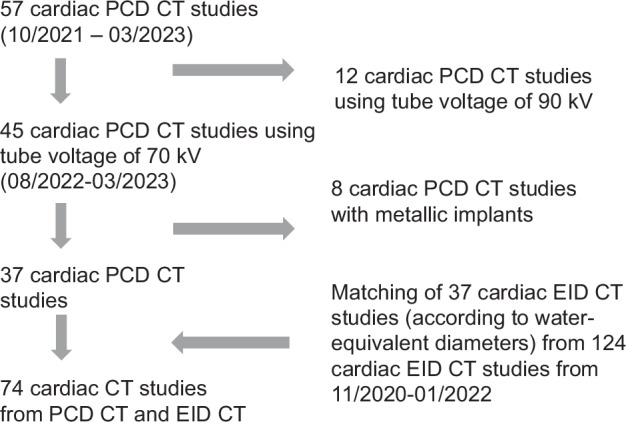
A flowchart of the study is presented, from initial retrieval to final study cohort

Figure 1 represents the flowchart of the study, from initial retrieval to final study cohort. Thirty-seven children examined on PCD CT and 37 matched children examined on EID CT were included. Demographic characteristics of the study cohorts are shown in Table 2. The PCD CT group included 13 girls and 24 boys, and the EID CT cohort included 18 girls and 19 boys. Age, height, weight, and body mass index did not significantly differ between the two study cohorts (Table 2). As the patient cohorts were matched based on their water-equivalent diameters, the water-equivalent diameters were comparable between the two groups (Table 2).

**Table 2:** Patient characteristics

	Cohort PCD CT, *n* = 37	Cohort EID CT, *n* = 37	*p*-value
Female/male	18/19	13/24	
Age (days) Median age (days)	794 ± 1016 190	815 ± 957 254	0.76^#^
Height (cm)	77 ± 27	79 ± 27	0.32^+^
Weight (kg)	11.6 ± 9.3	11.4 ± 10.6	0.86^#^
BMI (kg/m^2^)	14.6 ± 2.6	14.5 ± 2.9	0.74^#^
Water-equivalent diameter (mm)	140.6 ± 28.6	141.0 ± 28.1	0.20^#^

Data are given as mean ± standard deviation

*PCD CT* photon-counting detector computed tomography, *EID CT* energy-integrating detector computed tomography, *BMI* body mass index

^#^ Wilcoxon signed rank tests were performed for the analysis

^+^ Paired *t*-tests were used for the analysis

### Radiation exposure

**Fig. 2:**
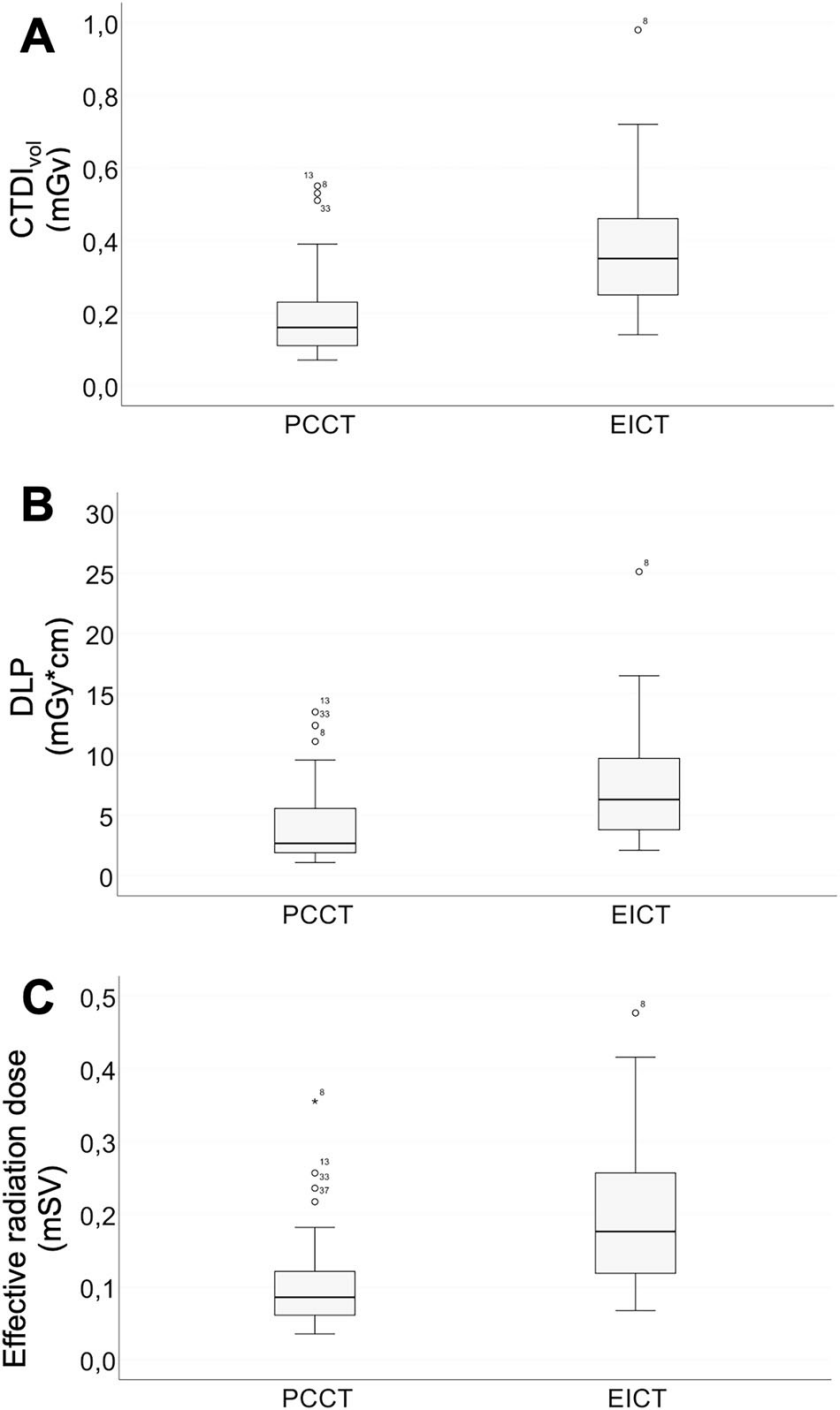
Box and whisker plots compare photon-counting detector computed tomography (PCD CT) and energy-integrating detector computed tomography (EID CT) examinations (*n* = 37 each) in terms of measure of volume CT dose index (CTDI_vol_; **A**), dose length product (DLP; **B**) and effective radiation dose (**C**). The middle bars in the boxes indicate the median; the whiskers are the lower and upper quartiles. Nonoutlier range includes values within 1.5 × interquartile range; outliers are values beyond the nonoutlier range. PCD CT has significantly lower CTDI_vol_, DLP, and effective radiation dose compared to EID CT (*p* < 0.001)

**Fig. 3:**
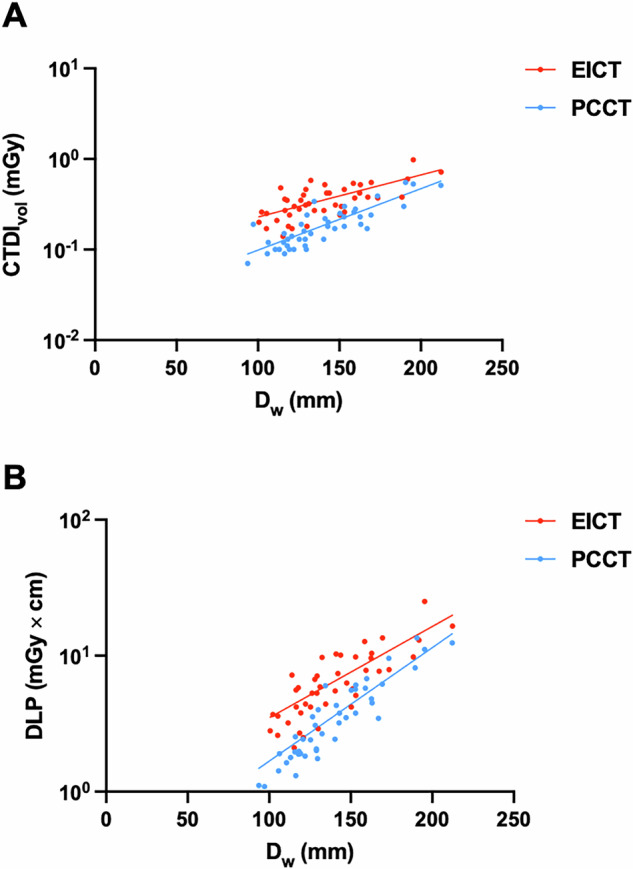
Regression analyses of volume CT dose index (CTDI_vol_; **A**) and dose length product (DLP; **B**) plotted against water-equivalent diameter (Dw; *n* = 37 each). Lower scattering of photon-counting detector computed tomography (PCD CT) compared to energy-integrating detector computed tomography (EID CT) data points was reflected by higher R-squared values of PCD CT (CTDI_vol_: 0.75 versus 0.51; DLP: 0.83 versus 0.65)

Volume CT dose index and dose length product were significantly lower on PCD CT (0.20 ± 0.12 mGy and 4.06 ± 3.22 mGy*cm) compared to EID CT (0.37 ± 0.17 mGy and 7.21 ± 4.67 mGy*cm; Wilcoxon signed rank tests*, p* < 0.001; see Table 3). Therefore, the mean CTDI_vol_ was reduced to approximately 46% and the mean DLP to approximately 44% with the PCD CT system. The effective radiation dose was also significantly lower on the PCD CT system compared to the EID CT scanner (0.11 ± 0.70 mSv versus 0.20 ± 0.11 mSv*, p* < 0.001, Table 3). Figure 2 presents box and whisker plots for CTDI_vol_, DLP, and effective dose in comparison of the PCD CT versus the EID CT scanner. Figure 3 demonstrates regression analyses of CTDI_vol_ and DLP plotted against water-equivalent diameter. In children examined on PCD CT, a lower scattering of radiation dose was determined with higher R-squared values of PCD CT versus EID CT (CTDI_vol_: 0.75 versus 0.51; DLP: 0.83 versus 0.65). Moreover, Fig. 3 shows that the differences in radiation exposure tend to be higher in children with lower water-equivalent diameter, i.e., smaller children.

**Table 3:** Differences in radiation dose, quantitative, and qualitative image quality between PCD CT and EID CT examinations

	PCD CT examinations, *n* = 37	EID CT examinations, *n* = 37	*p*-value
CTDI_vol_ (mGy)	0.20 ± 0.12	0.37 ± 0.17	< 0.001
DLP (mGy*cm)	4.06 ± 3.22	7.21 ± 4.67	< 0.001
Effective radiation dose (mSv)	0.11 ± 0.70	0.20 ± 0.11	< 0.001
SNR	6.63 ± 3.73	6.41 ± 4.42	0.80
CNR	18.67 ± 10.57	19.38 ± 13.15	0.72
Beam-hardening artifacts	13.16 ± 10.68	12.45 ± 7.87	0.86
Homogeneity of contrast	3.97 ± 0.44	3.89 ± 0.77	0.55
Sharpness	3.35 ± 0.72	3.57 ± 0.69	0.17
Pulsation artifacts	3.57 ± 0.77	3.73 ± 0.61	0.33
Overall image quality	3.73 ± 0.56	3.73 ± 0.51	1.00
Image quality of LM	3.92 ± 1.06	4.14 ± 1.03	0.36
Image quality of proximal LAD	3.78 ± 0.98	4.11 ± 1.02	0.14
Image quality of proximal LCX	3.22 ± 1.06	3.49 ± 1.15	0.31
Image quality of proximal RCA	3.51 ± 1.17	3.68 ± 1.06	0.57

Data are given as mean ± standard deviation if not indicated differently. Wilcoxon signed rank tests were performed for all analyses

*PCD CT* photon-counting detector computed tomography, *EID CT* energy-integrating detector computed tomography, *CTDI*_*vol*_ volume CT dose index, *DLP* dose length product, *SNR* signal-to-noise ratio, *CNR* contrast-to-noise ratio, *LM* left main artery, *LAD* left anterior descending artery, *LCX* left circumflex artery, *RCA* right coronary artery

### Image quality

**Fig. 4:**
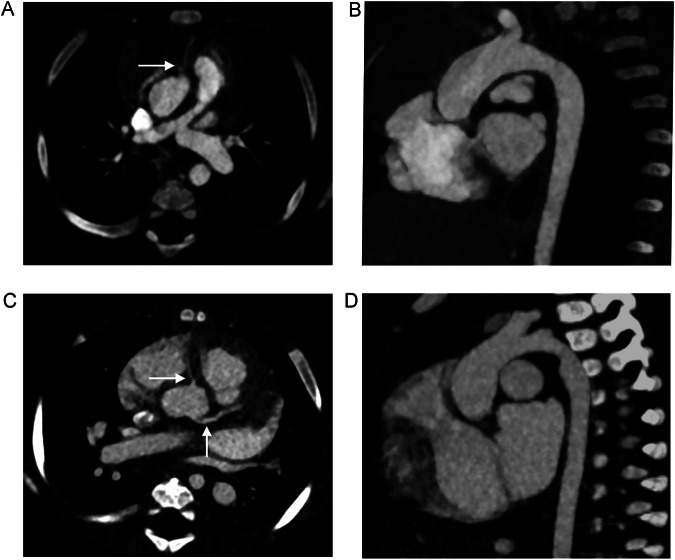
**A**, **B** Cardiac examination on photon-counting detector CT of a 7-month-old female patient, born with tetralogy of Fallot. Volume CT dose index (CTDI_vol_) of this examination was 0.11 mGy, dose length product (DLP) was 1.9 mGy*cm, water-equivalent diameter was 118 mm. **C**, **D** Cardiac examination on energy-integrating detector CT of a 5-month-old female patient, born with atrioventricular septal defect. CTDI_vol_ of this examination was 0.35 mGy, DLP was 5.8 mGy*cm, water-equivalent diameter was 118 mm. The cardiac anatomy can be very well depicted in both studies, including the coronary arteries (arrows in the axial orientations **A** and **C**). **B** and **D** show the aortic arch in parasagittal orientation

No significant difference between the two patient groups was seen for the quantitative image quality parameters. Results of the calculation of SNR, CNR, and beam-hardening artifacts are presented in Table 3. Additionally, no significant differences were found in the qualitative assessment of image quality for homogeneity of contrast, sharpness, presence of pulsation artifacts, and overall image quality (see Table 3 and Fig. 4). Moreover, the delineation of the proximal coronary segments did not significantly differ between the two scanners. Results from the quantitative and qualitative image assessment are summarized in Table 3.

## Discussion

This study demonstrates the possibility to reduce radiation exposure in cardiac CT of children by more than 40% without impairing quantitative nor qualitative image quality. Furthermore, typical artifacts that appear in CT protocols with lower radiation doses, such as beam hardening [36], were not increased by using PCD CT compared to EID CT scanner.

Pediatric patients with congenital heart disease are often less than 3 years of age and therefore very vulnerable to radiation exposure. At the same time, exact depiction of the cardiac anatomy is of incremental value for interdisciplinary planning of the therapeutic strategy; therefore, precise evaluation and description of small anatomic structures are necessary. In our study, despite the significantly reduced radiation dose of PCD CT examinations, qualitative assessment of proximal coronary arteries remained comparable to studies from the EID CT system (Table 3). This is in line with recently published results on adult cardiac CT examinations on a PCD CT system with excellent depiction of coronary artery stenosis [37] as well as coronary stents [38]. A recent study by Dirrichs et al [23] reported that PCD CT offered higher SNR and CNR than EID CT in children suspected of having cardiac defects when comparing CT studies using tube voltage of 90 kV (PCD CT) versus 70 kV (EID CT). One of the main differences between our study in comparison to the study by Dirrichs et al [23] is the radiation dose used for cardiac CT: Dirrichs et al [23] reported a mean DLP of 14.34 mGy*cm on PCD CT and 16.14 mGy*cm on EID CT examinations in children [23]. In our study, mean DLP on PCD CT was 4.06 mGy*cm and 7.21 mGy*cm on EID CT system. Therefore, we only used one third of the radiation dose compared to the mentioned study, despite including an older patient collective (mean/median age in our study 794/190 days on PCD CT and 815/254 days on EID CT versus median age of 48 days on PCD CT and 77 days on EID CT in the publication of Dirrichs et al [23]). The strength of our study is the matching of the patient cohorts, which were examined on PCD CT versus EID CT systems, and the technically adapted CT protocols using a tube voltage of 70 kV on both scanners.

Recent publications found excellent image quality for PCD CT examinations in children. Radiation dose values, comparable to our study, with good to excellent image quality were reported from a recent study describing initial experiences with low-dose, unenhanced chest PCD CT imaging in children with CTDI_vol_ of 0.21 ± 0.08 mGy and DLP of 4.93 ± 2.27 mGy*cm [39]. The assessed radiation exposure corresponds to published values, where CTDI_vol_ of 0.2 ± 0.08 mGy is defined as ultra-low dose chest CT imaging in children [40]. One of the main limitations when using low radiation doses in CT protocols on EID CT systems is the impairment of image quality and the increase of artifacts, such as beam hardening [36]. In our study, image artifacts did not increase when using very low radiation doses with PCD CT compared to EID CT. Similar results have been reported before for adult patients: Niehoff et al [41] observed that the radiation dose could be significantly reduced in patients who underwent ultra-low-dose PCCT examination for the detection of urolithiasis without compromising image quality and diagnostic confidence. Furthermore, Symons et al [29] compared PCD CT versus EID CT studies using a lung cancer screening protocol (120 kV, 20 mAs) with an iterative reconstruction algorithm for the examination of 30 healthy volunteers. Quantitative measurements showed better image quality on PCD CT compared to EID CT studies in areas prone to beam hardening, for example, the apical lobes and around the vertebrae, with 15–17% lower image noise and 21% higher lung nodule CNR [29]. To conclude, PCD CT imaging has been shown to be a very good compromise between increased spatial resolution, decreased radiation dose, and equivalent or reduced image noise. The reduction of > 40% radiation exposure did not result in decreased image quality in our study; however, the *p*-value of 0.08 for the delineation of the proximal LAD (Table 3) with tendentially higher values in EID CT examinations might indicate that further lowering of the radiation exposure on PCD CT might impair image quality.

In our study, no significant differences for quantitative image analysis (SNR, CNR, and beam-hardening artifacts) were found between images from EID CT and PCD CT examinations. Higher CNR with PCD CT has been reported in several studies in various body parts [42–44]. Nevertheless, Gutjahr et al [45] could show in their study when comparing EID CT and PCD CT studies at clinical dose rates in human cadavers that CNR on PCD CT examinations increased relative to the EID CT of 11%, 23%, 31%, and 38% at 80, 100, 120, and 140 kV levels, respectively. In our study, a tube voltage of 70 kV was used; therefore, differences might be too small to be significant. Stålhammar et al [46] examined 35 children with congenital heart defects at 70 kV and 35 children with congenital heart defects at 90 kV on PCD CT system; size-dependent dose estimate and effective dose were higher on 90 kV, whereas qualitative diagnostic quality was rated equally between the two tube voltages [46]. Jungblut et al [47] compared the image quality of PCD CT versus EID CT examinations using an anthropomorphic chest phantom containing 14 pulmonary nodules. With a dose-matched volume CT dose index of 0.41 mGy, the sensitivity to detect nodules was 95% on PCD CT versus 86% on EID CT examinations [47]. Nevertheless, most studies used dose-matching for their study participants, while in our study, patients were matched according to water-equivalent diameters, and radiation doses were significantly different between the two scanners. Furthermore, we compared results from the PCD CT system with CT studies from the latest generation dual-source EID CT. Knowing that image quality can be improved using similar radiation doses while image quality remains comparable to conventional CT, this knowledge can allow improvement of imaging in patients, where very low dose is desirable, such as in pediatric imaging.

This study has limitations. First, the cohort size is limited, which is due to the matching of the two patient cohorts on PCD CT versus EID CT. However, to our knowledge, this is the largest published patient cohort with congenital heart disease that underwent cardiac PCD CT using a tube voltage of 70 kV. We opted for fast, high-pitch helical scans without ECG-gating to minimize examination time, including sedation. According to our results, even small vessels, as the proximal segments of the coronary arteries, were depicted precisely in a high amount. ECG-gating might further enhance image quality. However, according to our results, fast high-pitch helical scans without ECG-gating might be suitable for the patient collective with congenital heart disease. However, the patients suffered from different forms of congenital heart defects. Despite this, the examinations were comparable, as all CT scans were started at the time point when all four heart chambers were optimally filled by contrast medium. The technical protocols of the CT scanners were adapted as clinically advisable and technically feasible, with only slight differences, such as matrix and pitch. One important advantage of CT examinations in children with congenital heart disease lies in their ability to provide highly detailed anatomical visualization. However, functional information, such as that obtained through magnetic resonance imaging, is lacking. Another important limitation of our study is that we excluded patients with metallic devices, which limits our patient collective to a restricted subgroup since the prevalence of metallic devices in patients with congenital heart disease is relatively high. Further research is needed, including data from these examinations. Nevertheless, in this present study, we aimed to analyze comparable groups of patients and to be as precise as possible in terms of patient matching. Another limitation is the retrospective design. Still, we included all consecutive patients that underwent cardiac CT on PCD CT and matched patients from EID CT according to water-equivalent diameters, resulting in comparable groups with no significant differences in age, weight, height, or BMI. Reducing radiation exposure by using PCD CT while preserving or enhancing image quality might substantially advance cardiac imaging in children.

In summary, we could show that image quality of cardiac PCD CT examinations in children remained stable with a reduction of radiation exposure of more than 40% compared to CT studies from EID CT. PCD CT for imaging of congenital heart disease in infants and children is therefore not only feasible but also advisable.

## Abbreviations

BMI: Body mass index
CNR: Contrast-to-noise ratio
CT: Computed tomography
CTDI_vol_: Volume CT dose index
DLP: Dose length product
ECG: Electrocardiogram
EID CT: Energy-integrating detector CT
LAD: Left anterior descending artery
LCX: Left circumflex artery
LM: Left main artery
PCD CT: Photon-counting detector CT
ROI: Region of interest
SD: Standard deviation
SNR: Signal-to-noise ratio
